# Assistive technology enables inclusion in higher education: The role of Higher and Further Education Disability Services Association

**DOI:** 10.4102/ajod.v8i0.558

**Published:** 2019-08-22

**Authors:** Marcia Lyner-Cleophas

**Affiliations:** 1 Disability Unit, Centre for Student Counselling and Development, Stellenbosch University, Stellenbosch, South Africa

**Keywords:** disability inclusion, assistive technology, post-school education and training sector (PSET), Disability Units, HEDSA, listserv, MapAbility

## Abstract

**Background:**

Using assistive technology is one way to foster inclusion of students in the post-school education and training (PSET) sector.

**Objectives:**

Higher and Further Education Disability Services Association (HEDSA) enables the sharing of new knowledge about assistive technologies through its symposia, and making information available on its website. Additionally, it facilitates dialogue and collaboration amongst institutions in the PSET network using a listserv and newsletters, given that PSET institutions are spread countrywide.

**Method:**

This is an article based on a presentation at the 5th African Network of Evidence-to-Action in Disability (AfriNEAD) conference in Ghana in 2017 that focused on the value of assistive technology for students pursuing studies in the PSET sector and the role played by HEDSA in South Africa.

**Results:**

The positive gains and existing gaps in disability inclusion in the higher education sector in South Africa are highlighted, with reference to access to technology. All higher education institutions have internet access and can thereby make use of listservs to communicate information. MapAbility is a way that prospective students can gain a snapshot view of available resources at institutions of learning, using the internet.

**Conclusion:**

An association such as HEDSA plays a critical role in the PSET sector to enhance disability inclusion using online tools to disseminate information.

## Introduction

South Africa is in the process of healing from sustained exclusion and discrimination, segregation, colonisation, racism and many other social inequalities and injustices that have become ingrained in its society. Class, gender, race and disability have borne the brunt of much of the oppression. Where students with disabilities were in educational settings, they were further segregated into special schools and mainstream schools (Naicker 2005). Still further segregation occurred within some of the mainstream schools where there were special classes (Howell & Lazarus 2003; Swart & Pettipher 2011). Special schools still exist, but broader options are in place, given inclusive education, where more students have access to receiving matric certificates and are furthering their education in the post-school education and training (PSET) sector, which is one visible way in which disability inclusion is broadening.

International and national treaties and policies point to a human rights-based approach that eliminates discrimination, with specific reference to people with disabilities. These include the Constitution of the Republic of South Africa (Republic of South Africa 1996); the South African National Development Plan (NDP) (2030) (Republic of South Africa 2013); the United Nations Convention on the Rights of Persons with Disabilities (United Nations 2007); the Strategic Disability Policy Framework in the PSET system (Republic of South Africa 2018), which is the latest disability policy in the Department of Higher Education and Training (DHET); and the *Promotion of Equality and Prevention of Unfair Discrimination Act* 4 of 2000 (PEPUDA) (Republic of South Africa 2000). Discrimination has existed for hundreds of years. South Africa is now in her 25th year of democracy and striving towards a more inclusive society that acknowledges diversity and its value. The striving towards social justice through many ways continues to be a long struggle. However, the need to be organised and focused as a nation striving to be inclusive in all respects is critical to a socially inclusive society. According to Bell (2016), social justice refers to the process of recreating societal principles in a way that echoes equity, recognition and inclusion. Given the history of South Africa, establishing social justice will be a long process as we review and reflect on our practices and thinking about people and human rights. This policy environment and the quest for social justice pave the way for acknowledging and including diversity in people. Increasingly, the use of assistive technology does foster inclusion, with specific reference to disability inclusion, as this article shows.

## Disability inclusion practices in the post-school education and training sector

Disability inclusion means that we acknowledge disability as part of the diversity in people, thereby enhancing greater interaction and participation in society and educational systems. We remain aware of social injustices, including how we exclude people based on disability, thereby acting in ableist ways. Ableism becomes a way of subjugating people merely because they do not present in normative ways. Ostiguy, Peters and Shlasko (2016:299) view ableism as ‘disability oppression’ and as a ‘pervasive system that oppresses people with disabilities while privileging people who do not currently have disabilities’. In the PSET sector (education and training beyond basic education, such as colleges and universities) we need to be cognisant of how we might be consciously or unconsciously excluding people from educational opportunities.

The latest policy guideline of the DHET for the post-school sector is called the Strategic Disability Policy Framework in the PSET system (Republic of South Africa 2018). This Strategic Disability Policy Framework acknowledges the need for disability inclusion in post-school education and has put in place this framework to accelerate access and success for people with disabilities. This is the first definitive framework in place for this sector and augurs well for further disability inclusion to take place for students with disabilities leaving high school to further their education.

Disability, however, is intertwined in matters relating to class, race, gender and economics. Affordability and access to support can become exclusionary depending on a combination of these factors. The rise of technology and assistive technologies has impacted how we become socially and economically included. It has also played a life-changing role in access to education for everyone, particularly to people with disabilities. With this access to technology also comes possible exclusion, especially in low-tech environments where access is poor.

Students in the PSET sector have much reading material to work through. More options regarding how to access reading and learning materials, in addition to Braille and Sign Language, are examples. Assistive technologies such as the use of screen-reading software to access reading material as well as text enlargement software have enhanced the access to information and learning material. Although assistive technologies have been a facilitator of inclusion and participation in living and learning environments, access to technology has mixed spread and application, particularly in Africa, as surveyed in a few countries, that is South Africa, Namibia, Malawi and Sudan (Visagie et al. 2016). Amongst others, their findings were that within country access to assistive technologies differs and that governments need to play a greater role where affordability and access to technology are compromised. In the PSET sector, given the high volumes of reading material, technology plays a critical role in being an enabler and support to people to ensure higher education levels with the resulting improved successful participation in the economy.

## The role of Disability Units as enablers of inclusion

Disability Units or centralised support services in the PSET sector have steadily been put in place on most of our campuses in South Africa. However, these should not be seen as the only and main stakeholder in education (Mutanga 2017). These units, referred to as Disability Rights Units in the Strategic Disability Policy Framework (Republic of South Africa 2018), but also called by various names at our institutions, have been required to facilitate access to a range of students with disabilities on our campuses. Although this DHET framework admits that Disability Units are often seen as being on the fringes in institutions and not integral, they acknowledge their critical facilitative role. Some of the expectations of these units have been unrealistic, particularly given the resource constraints, as noted in the Foundation of Tertiary Institutions of the Northern Metropolis (FOTIM 2011) report and by Howell (2005). Such constraints result in either no services, or minimal support or a blurring of services and roles, such as being responsible for professional Sign Language Interpretation Services on campus for deaf students. The Disability Units’ role should mostly be a facilitative and advisory one in instances where the Disability Unit does not have the expertise and resources. Professional Sign Languages services, for instance, are best left in the language services domain in the PSET sector given its specialist nature, but always in collaboration with disability services.

Disability Units could liaise with state services and product suppliers to enable students to obtain the required assistive devices, such as hearing devices or assistive software for computers. Disability Units can also liaise with bursary providers to award bursaries to their students. Liaising with stakeholders on campus from top management and other support to academic departments and their staff is key to successful partnerships between students and support staff, especially where assistive technologies can be provided. Disability Units therefore play a key role in facilitating support such as assistive technologies and are necessary links to service providers within and outside the PSET setting. Most Disability Units are members of Higher and Further Education Disability Services Association (HEDSA) and use the HEDSA network to improve their knowledge of assistive technologies.

## Assistive technology fosters disability inclusion

The value of assistive technologies in education as facilitators of access to information, access to website material and access to learning materials cannot be underestimated, as we strive towards social justice in South Africa and disability inclusion in the educational setting in particular.

The availability of technology has made access to information increasingly easier (Duplaga 2017). This is proving to be an inclusive tool that allows for a variety of users to access information online, amongst other benefits. Assistive technology has enabled more forms of making inaccessible information more accessible. Examples would be a screen reader such as Openbook, Magic, Job Access with Speech (JAWS)^1^ or NonVisual Desktop Access (NVDA)^2^ and ZoomText (the latter enlarges texts). Other assistive software could be Read & Write, reader pens such as the C-pen, Dictaphones and WYNN, which assist students who have difficulties in reading and writing. These could be purchased by Disability Units for the use of students but are better placed in the computer labs that students use to access their learning and reading material. It is useful to have such software on students’ personal devices such as laptops as they can then access their information when they are off campus too. Personal assistive devices such as hearing aids and walking sticks are aids that would be purchased usually by students for their personal use.

In low-tech environments, given the varied access to resources, students can access free open-source software such as NVDA. Electronic books can be accessed from certain publishers provided multiple copies are not made and given to students who do not have disabilities. Cell phones can also be used as recording devices in the absence of pricier assistive technologies.

Online platforms have bridged many communication gaps. Meetings can be held across the globe. In the educational context, information, information communications technologies (ICTs) and assistive technologies create good opportunities for universal access in instruction and courses (Burgstahler 2015). Information communications technologies can lower barriers in an educational setting. It is also described as creating an environment that can broaden access and improve collaboration and networking in the educational setting, as indicated in the latest Strategic Disability Policy Framework (Republic of South Africa 2018). This policy seeks to develop an environment in which norms and standards can be set in the PSET sector in South Africa. Although the advent of technology and assistive technologies has made huge positive differences in the lives of people, often people with disabilities are faced with high costs of technologies amidst high unemployment rates (Atkinson & Castro 2008; Visagie et al. 2016), inadvertently excluding them.

Duplaga (2017) conducted studies, which indicate that email communication is mostly used in the digitised world. Searching the Internet for information also indicated very high usage. Both of these are used by all people, but especially for people with visual or physical disabilities, access to information is easier.

## The role of Higher and Further Education Disability Services Association

In South Africa, 26 higher education institutions (HEIs) and 50 technical and vocational education training sector (TVETs) have been established as non-privatised institutions in the PSET sector. Most of the institutions have some kind of student support office in place, and most of the 26 HEIs are members of HEDSA. Fewer TVETs are HEDSA members at this stage. Higher and Further Education Disability Services Association arose out of a need of FOTIM, where practitioners in disability support met in a selected region in South Africa, to have disability matters organised broadly across all of the provinces in South Africa. It is the first and only organisation of its kind in South Africa that organises disability support across Disability Units and likewise stakeholders in the PSET sector.

Higher and Further Education Disability Services Association has various roles in the PSET sector, including how it advises as a community of practice on the assistive technologies across PSET institutions. Higher and Further Education Disability Services Association was established in October 2006 and is a community of practice for members of HEDSA who work in the PSET sector, primarily in disability support services. It is a non-profit organisation (NPO) registered with the Department of Social Development. Its website address is http://www.hedsa.org.za. Its main funder is the Carl & Emily Fuchs Foundation.

In addition to the main funder, institutions have an annual subscription of R1500 ($102, 13 on 02 August 2019). It holds a biennial general meeting where a new executive is elected for 2 years. The executive coordinates activities and projects nationally and mainly uses technology to do this. Members also work as Disability Unit staff at the various PSET institutions countrywide (mainly from HEIs).

Higher and Further Education Disability Services Association has eight core objectives, which are to: stimulate dialogue, promote rights, network & cooperate, facilitate inclusivity, support advocacy, identify needs, undertake projects and encourage collaboration. Given that HEDSA is not an organisation with a physical address with staff located in a specific place, it operates in the virtual space and is heavily reliant on technology. It operates by email mostly and has presence through a website.

**FIGURE 1:**
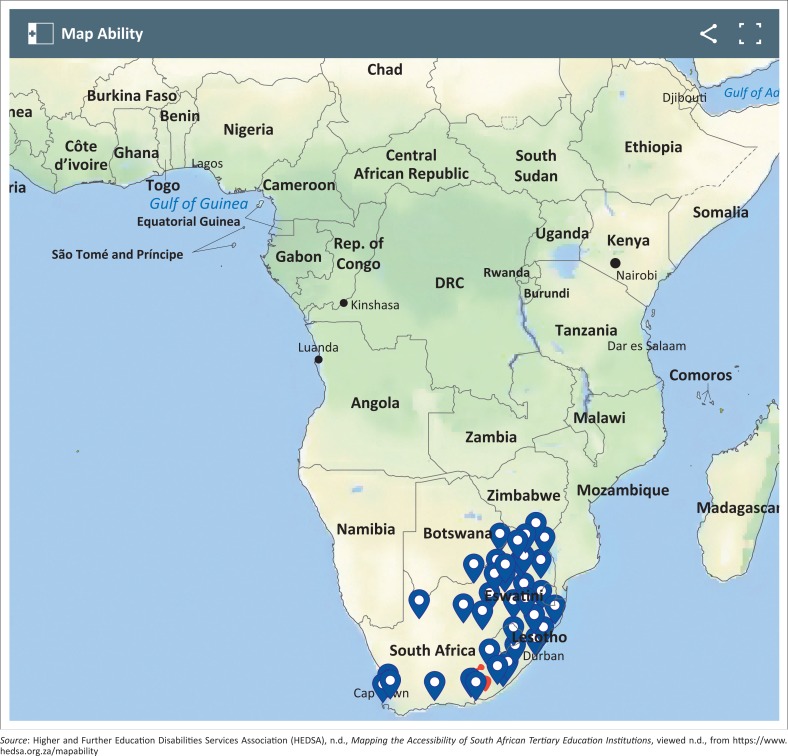
MapAbility in South Africa.

The value of HEDSA lies in its sharing of information across institutions, to establish best practice for specific institutions given their realities. To share information, the two innovative ways that it has grown are by using a ‘listserv’ as well as MapAbility (Figure 1) – both are means of communicating information via its membership network, as part of its collaboration mandate.

A listserv was actively started in 2018 by using the Google platform hedsa-community@googlegroups.com. The members of HEDSA can request to be part of the listserv. The listserv enables PSET institutions to share practices of inclusion. Questions and information shared include guidelines about test and exam concessions, the use of service dogs in the PSET sector, institutional versus individual responsibility for the purchase of assistive technologies and the kinds of assistive technologies used in institutions, to name a few of the ideas that are exchanged on a platform that tracks and stores useful information.

In addition to the listserve, HEDSA established an online platform that allows prospective students to check whether the institutions that they intend to apply for have the kinds of access services and support that they would need, should they be accepted to study at the specific institution. This online mapping platform is called MapAbility. MapAbility aims to provide students with online information about accessibility in the PSET sector on a map of Africa. This guides them regarding accessibility in the location that they wish to attend or visit. The International Exchange Erasmus Student Network started such an online mapping site to assist students with disabilities with accessibility information^3^. MapAbility in South Africa is the first of its kind on the continent and promises to guide students and staff regarding accessibility in the PSET sector.

The website of HEDSA also directs people such as students and staff to service providers of assistive technology^4^. At the biennial symposium held by HEDSA, there is an opportunity for assistive technology providers to exhibit their technologies to the attendees. Many contacts are built on this PSET platform, and good collaborations are formed.

## Conclusion

Social justice cannot be achieved without the inclusion of all people, including people with disabilities, in educational settings. The human rights-based approach is entrenched in various national and international policies and treaties. Policies, guidelines, intention and rhetoric must be enacted and put into practice. Economic constraints are a reality, but free open-source software such as NVDA can be used as screen-reading software for people with difficulties in reading and writing in low-resource environments. At the PSET institutions in South Africa, it is important to network across institutions to improve institutional knowledge and support to staff and students, with reference to ways in which barriers to learning can be overcome. Higher and Further Education Disability Services Association creates an online opportunity for enhanced information sharing and collaboration through its listserv, symposia, the online MapAbility tool and the captive audience it has with the community of Disability Unit staff. The staff are diverse with a range of expertise and skills that can be shared across Disability Units to enrich practice. Institutions in South Africa that are part of the PSET sector would find it useful to add the ways in which they are inclusive at their institutions, to an online tool such as MapAbility. This presents a quick guide to services at TVET colleges or HEIs. The HEDSA website can be contacted regarding mapping their services on MapAbility.

The listserv, given that emails are very common today, is a useful tool to use and link with TVET colleges and HEIs, where quick questions need to be asked and multiple inputs are gathered as good practices for the various TVET colleges and HEIs.

Technical and vocational education training sectors are encouraged to join HEDSA, as well as HEIs that have not yet done so. It offers a community of practice through which to share information and innovative ideas and attend affordable biennial symposia. As an organisation it makes input into government policies as needed and requested.

## Footnotes

1 JAWS. https://www.freedomscientific.com/Products/Blindness/JAWS.

2 https://www.nvaccess.org/.

3 https://esn.org/students; https://www.politico.eu/article/the-mapability-project-taking-the-initiative-on-mobility/.

4 https://www.hedsa.org.za/technology.
